# Draft Genome Sequence Analyses of Two Novel *Marinobacter suadae* sp. nov. and *Wenyingzhuangia gilva* sp. nov. Isolated from the Root of *Suaeda japonica* Makino

**DOI:** 10.3390/life14030296

**Published:** 2024-02-22

**Authors:** Sunho Park, Inhyup Kim, Geeta Chhetri, Yonghee Jung, Haejin Woo, Taegun Seo

**Affiliations:** Department of Life Science, Dongguk University-Seoul, Goyang 10326, Republic of Korea; eksvnd97@dgu.ac.kr (S.P.); duckling91@dgu.ac.kr (I.K.); lucky_salman@dongguk.edu (G.C.); joh2395@dongguk.edu (Y.J.); woohj999@dongguk.edu (H.W.)

**Keywords:** pangenome, whole genome, *Suaeda japonica* Makino, genomic, *Marinobacter*, *Wenyingzhuangia*, Up-to-Date Bacterial Core Gene set

## Abstract

Gram-negative, rod-shaped, and aerobic bacteria designated chi1^T^ and chi5^T^ were isolated from the root of *Suaeda japonica* Makino. Phylogenetics utilizing 16S rRNA and whole-genome sequences of the two novel strains chi1^T^ and chi5^T^ confirmed that they were related to the genera *Marinobacter* and *Wenyingzhuangia*, respectively. For the novel strains chi1^T^ and chi5^T^, the digital DNA–DNA hybridization values (19–20% and 22.1–36.6%, respectively) and average nucleotide identity values (74.4–76.5% and 79.1–88.9%, respectively) fell within the range for the genera *Marinobacter* and *Wenyingzhuangia*, respectively. Pangenome analyses of the novel strains chi1^T^ and chi5^T^ revealed 357 and 368 singletons genes, respectively. The genomic DNA G + C contents of the strains chi1^T^ and chi5^T^ were 57.2% and 31.5%, respectively. The major fatty acids of strain chi1^T^ were C_12:0_, C_16:0_, and summed feature 3 (C_16:1_ *ω*6*c* and/or C_16:1_ *ω*7*c*), while those of the strain chi5^T^ were iso-C_15:0_ 3OH, iso-C_17:0_ 3OH, and iso-C_15:0_. Data from the phylogenetic, phylogenomic, pangenome, genomic, physiological, and biochemical analyses indicated that the novel strains were distinct. Therefore, we propose the names *Marinobacter suadae* (type strain chi1^T^ = KACC 23259^T^ = TBRC 17652^T^) and *Wenyingzhangia gilva* (type strain chi5^T^ = KACC 23262^T^ = TBRC 17900^T^) for the studied bacterial strains.

## 1. Introduction

*Suaeda japonica* Makino (Chenopodiaceae) is an annual dyeing plant distributed in the tidal flats of the Yellow Sea in the Republic of Korea and Kyushu in Japan. It grows up to 20–50 cm in length [1]. Moreover, *S*. *japonica* species have been used as functional foods and medicinal plants because of their beneficial effects, including antioxidant, antidiabetic, and α-glucosidase-inhibitory effects [2]. In this study, the novel strains *M. suaedae* chi1^T^ and *W. gilva* chi5^T^ were isolated from *S*. *japonica* Makino in a unique ecological environment that was between marine and terrestrial environments. In this unique habitat, tidal flats are known to contain abundant invaluable biological resources that play a pivotal role in the restoration, stabilization, and nutritional cycling of coastal ecosystems [3,4,5,6].

The genus *Marinobacter* was first proposed by Gauthier et al. in 1992 [7], with the type species being *M*. *hydrocarbonoclasticus*. This genus belongs to the class *Gammaproteobacteria* in the order *Pseudomonadales* in the family *Phyllobacteriaceae*. The genus *Marinobacter* consists of 60 species at present, and several child taxa have been validly published (https://lpsn.dsmz.de/genus/marinobacter accessed on 1 November 2023) [8]. *Marinobacter* is a metabolically flexible genus that inhabits a very wide range of marine, tidal, and sediment environments [7,9,10,11,12,13,14,15,16,17]. These bacteria are mostly heterotrophic; however, in some cases, they are mixotrophic [9,18,19]. *Marinobacter* species are halophilic or halotolerant bacteria that have adapted to survive in a wide range of salinity concentrations [7,10,15]. They are thus an interesting group of microorganisms that can be used as a source of salinity-adapted enzymes [20]. The presence or absence of polar flagella in the genus *Marinobacter* depends on the salt concentration [7]. In addition, some species [7,12] are recognized for their ability to degrade polycyclic aromatic hydrocarbons (PAHs).

The genus *Wenyingzhuangia* was first proposed by Liu et al. in 2014 [21], with the type species being *W*. *marina*. This genus belongs to the class *Flavobacteria*, order *Flavobacteriales*, and family *Flavobacteriaceae*. The genus *Wenyingzhuangia* consists of five species at present, and several child taxa have been validly published (https://lpsn.dsmz.de/genus/wenyingzhuangia, accessed on 2 June 2023) [8]. The five *Wenyingzhuangia* species were isolated from seawater [22,23], a tidal zone at the estuary [24], the red alga *Gracilaria vermiculophylla* [25], and the water of a culture tank of a recirculating mariculture system [21]. After comparing the phylogenetic and genomic features of the novel strains chi1^T^ and chi5^T^, we designated them as genera *Marinobacter* and *Wenyingzhuangia*, respectively.

## 2. Materials and Methods

### 2.1. Isolation

Samples were collected from the root tissue of a *Suaeda japonica* colony in Seongmodo (Republic of Korea, 37°40′28.6″ N, 126°22′35.2″ E). Five *S*. *japonica* specimens were randomly collected during low tide, ranging from areas closer to the sea to those further inland.

The roots of each of the five *S*. *japonica* specimens were washed with sterile distilled water to remove dust and dirt, lightly washed in sterile 0.1% Tween 20 for 30 s, and washed twice in sterile distilled water. Subsequently, the root specimens were surface sterilized with ethanol (95%; Duksan; Ansan, Republic of Korea) for 5 min, followed by sodium hypochlorite (1%; Duksan; Ansan, Republic of Korea) for 5 min. The specimens were then washed thrice in sterile distilled water. The specimens were diluted with 10 mL sterile 0.85% NaCl solution while being rotated for 30 min in a 15 mL conical tube [26]. Finally, 100 μL of standard ten-fold serial dilutions (10^−1^, 10^−2^, and 10^−3^) were spread onto marine agar 2216 (Difco, Eybens, France) and incubated at 30 °C for 3 days [27].

### 2.2. 16S rRNA Phylogeny

The 16S rRNA gene sequence analysis was used to determine the phylogenetic position of the strains chi1^T^ and chi5^T^. The complete 16S rRNA gene sequences of the strains chi1^T^ and chi5^T^ were then analyzed by SolGent Co., Ltd. (Daejeon, Republic of Korea). The 16S rRNA genes of the strains chi1^T^ and chi5^T^ were amplified using the universal bacterial primer sets 518F and 805R [28] and 27F and 1492R [29] by SolGent Co., Ltd. (Daejeon, Republic of Korea), and then sequenced via SeqMan software (DNASTAR, version 5.0) and Chromas (Technelysium, version 2.6.6). The complete 16S rRNA gene sequences of the novel strains were registered in the NCBI GenBank (www.ncbi.nlm.nih.gov/, accessed on 9 April 2023) [30] and EzBioCloud (http://ezbiocloud.net, accessed on 9 April 2023) [31] databases. To construct the phylogenetic tree, the sequences were aligned using the ClustalW tool. Phylogenetic trees were generated using the maximum likelihood (ML) [32], maximum parsimony (MP) [33], and neighbor-joining (NJ) [34] algorithms performed in Molecular Evolutionary Genetics Analysis (MEGA, version X) software [35]. Bootstrap values were analyzed with 1000 replications [36] using Kimura’s two-parameter model [37]. Strains *Acidiferrobacter thiooxydans* m-1^T^ (AF387301) and *Flammeovirga aprica* AB247553^T^ (NBRC 15941) were used as outgroups for the novel strains chi1^T^ and chi5^T^, respectively.

### 2.3. Genomic Features

Genomic DNA was extracted using the TruSeq DNA PCR-Free Kit (Novogene) by Macrogen Co., Ltd. (Seoul, Republic of Korea) [38]. Whole-genome shotgun sequencing of the novel strains was performed following the Illumina HiSeq X platform (Illumina; San Diego, CA, USA), and the assembly was performed using the SPAdes version 3.15.0 de novo assembler [39]. Busco version 5.1.3 [40] was used to assess the completeness of the genome assemblies of the novel strains. Prokka version 1.14.6 [41] was used to identify the locations of protein-coding sequences, tRNA genes, and rRNA genes and to assess the de novo assemblies of prokaryotes. The CheckM bioinformatics tool (http://ecogenomics.github.io/CheckM, accessed on 28 July 2023) [42] was used to assess the completeness and contamination of the whole-genome shotgun sequences. The assembled draft genomes of the novel strains were deposited in the NCBI database by the NCBI Prokaryotic Genome Annotation Pipeline (PGAP) (www.ncbi.nlm.nih.gov/genome/annotation_prok, version 6.5, accessed on 19 July 2023 [43]. The genomic DNA G + C contents were assessed based on genomic data. Based on whole-genome shotgun sequences, Rapid Annotation using Subsystem Technology (RAST) [44] and antibiotics and Secondary Metabolite Analysis Shell (antiSMASH, version 7.0) were used to identify gene functions and secondary metabolites, respectively [45]. Orthologous clusters (OCs) of the strains chi1^T^ and chi5^T^ and closely related members of *Marinobacter* and *Wenyingzhuangia*, respectively, were compared using the Ortho Venn3 online tool on 6 October 2023 [46]. A genomic circular feature map was constructed using the CGView server on 9 September 2023 [47].

The whole-genome shotgun sequences of the novel strains and closely related strains were obtained from the NCBI assembly database and used as a reference for calculating the average nucleotide identity (ANI) and digital DNA–DNA hybridization (dDDH) values of the novel strains by the Orthologous Average Nucleotide Identity Tool (OAT) (version 0.90) in EzBioCloud [48] and the Genome-to-Genome Distance Calculator (http://ggdc.dsmz.de, version 3.0), respectively [49].

Carbohydrate-active enzyme (CAZyme) gene clusters were analyzed using a dbCAN-PUL search and dbCAN-sub majority voting in the dbCAN web server (version 12.0) [50]. CAZymes were categorized into auxiliary activities (AAs), carbohydrate esterases (CEs), carbohydrate-binding modules (CBMs), glucoside hydrolases (GHs), glycosyl transferases (GTs), and polysaccharide lyases (PLs) and subjected to ORF classification. The genome dataset was organized into clusterable groups using the Batch Create Genome Set (version 1.2.0) tool and individual genes were categorized into these genome clusters utilizing Add Genomes to GenomeSet (version 1.7.6). These clusters were analyzed to identify orthologous protein sequences using Build Pangenome with OrthoMCL (version 2.0). Furthermore, to determine the genomic intersections between the novel strains and closely related strains, the Pangenome Circle Plot (version 1.4.0) was used. This analysis categorized the pangenome into distinct sets of singletons, core genes, and non-core genes. Phylogenomic trees were constructed using the Up-to-Date Bacterial Core Gene set (UBCG) [51].

### 2.4. Physiology and Chemotaxonomy

The strains chi1^T^ and chi5^T^ were cultured on marine agar plates at 30 °C for 3 days to assess their physiological, taxonomic, and biochemical characteristics. The novel strains were cultured at 30 °C for 3 days on marine agar, tryptic soy agar (TSA; Difco, Eybens, France), Luria–Bertani agar (LB agar; Difco, Eybens, France), Reasoner’s 2A agar (R2A; MB cell, Seoul, Republic of Korea), and nutrient agar (NA; Difco, Eybens, France) to assess their optimal growth on different media. Moreover, the novel strains were cultured on marine agar plates for 3 days to assess their optimal growth at temperatures ranging from 4–45 °C (4, 10, 15, 20, 25, 28, 30, 35, 38, 40, and 45 °C). To determine the optimal growth rate of the novel strains under various culture conditions, the strains were cultured with different NaCl concentrations and at different pH ranges in marine broth (MB cell, Seoul, Republic of Korea) at 30 °C for 3 days. In particular, 3 mL aliquots of marine broth containing various concentrations of NaCl (0–16% at 1% *w*/*v* intervals) and having different pH ranges (4.0–12.0 at 1.0 pH unit intervals) were inoculated with 30 μL of marine broth containing the precultured strains chi1^T^ and chi5^T^.

Four buffers with a final concentration of 50 mM (NaH_2_PO_4_ buffer, pH 5.0; phosphate buffer, pH 6.0–8.0; Tris buffer, pH 9.0–10.0; and Na_2_HPO_4_-NaOH buffer, pH 11.0–13.0) [52] were used to maintain the pH of the medium. Next, the inoculated novel strains were cultured at 30 °C for 3 days, and cell growth was assessed by observing the absorption spectrum at 600 nm using a spectrophotometer (Multiskan GO, Thermo Fisher Scientific). The catalase and oxidase activities were assessed using 3% (*v*/*v*) aqueous hydrogen peroxide (H_2_O_2_) solution and 1% (*w*/*v*) tetramethyl-p-phenylenediamine (BioMérieux), respectively. To determine the Gram types of the novel strains, Gregersen’s rapid method procedure was followed [53]. The strain chi1^T^ was cultured in marine agar plates containing various NaCl concentrations (3.5, 9.0, and 15.0%) for 3 days at 30 °C to observe the presence or absence of salt-responsive flagella. To observe the size and morphology of the cells cultured onto marine agar plates at 30 °C for 3 days, negative staining was performed using 3% uranyl acetate reagent and then observed under a transmission electron microscope (TEM; JEM-1010; JEOL). To determine whether the cells could grow under anaerobic conditions, the novel strains were incubated at 30 °C for 14 days using a GasPak jar (BBL, Cockeysville, MD, USA); an oxygen-absorber strip (Mitsubishi Gas Chemical) was used to remove oxygen. The motility of the novel strains was assessed in marine broth containing 0.4% agar at 30 °C for 3 days. The ability of the strains to hydrolyze chitin (1%; Tokyo Chemical Industry Co., Ltd., Tokyo, Japan), casein (1% skim milk; Biopure, Cambridge, MA, USA), starch (1%; Sigma-Aldrich, Saint Louis, MO, USA), Tween 20 (1%; Biopure, Cambridge, MA, USA), Tween 80 (1%; Samchun, Seoul, Republic of Korea), and carboxymethyl cellulose (1%; Duksan, Ansan, Republic of Korea) was assessed on marine agar plates at 30 °C for 7 days according to the method described by Smibert and Krieg [54]. Other physiological characteristics and acid production ability were assessed using API 20NE and API 50CH kits following the manual (BIOMÉRIEUX Co., Ltd, Marcy-l’Étoile, France). protocol after culturing the novel strains and reference strains on marine agar plates under optimum growth conditions. Cellular fatty acids were acquired by saponification, methylation, and extraction, as described previously by Kuykendall et al. [55], after culturing the cells on marine agar plates at 30 °C for 3 days. The fatty acids were extracted using the Sherlock Microbial Identification System V6.01 (MIS, database TSBA6; MIDI Inc., Newark, DE, USA). Fatty acids accounting for more than 10% and less than 1% of the total fatty acid contents were considered major fatty acids and TR, respectively. Polar lipids were extracted using two different solvents (chloroform/methanol/water [*v*/*v*/*v*, 65:25:4] and chloroform/acetic acid/methanol/water [*v*/*v*/*v*/*v*, 80:15:12:4]) and separated using two-dimensional thin-layer chromatography (TLC) [56]. The TLC plates were separated in two directions and developed with 0.2% ninhydrin reagent (Sigma Life Science), 5% molybdophosphoric acid (Sigma-Aldrich), Zinzadze’s reagent (molybdenum blue spray reagent, 1.3%; Sigma Life Science), and 2.5% α-naphthol sulfuric acid to identify amino lipids (ALs), total lipids, phospholipids (PLs), and glycolipids (GLs), respectively.

## 3. Results and Discussion

### 3.1. Isolation

From the marine agar plates, a circular, light ochre, smooth colony, along with a circular, yellow, convex colony, were both harvested and subsequently cultured at 30 °C for 3 days on new marine agar plates. Pure cultures of the isolates were maintained at −80 °C in marine broth containing 25% (*v*/*v*) glycerol. The purified strains were designated as the strains chi1^T^ and chi5^T^, respectively, and were deposited in the KACC (23259^T^ and 23262^T^) and TBRC (17652^T^ and 17900^T^) for systematic research.

### 3.2. 16S rRNA Phylogeny

The results of the 16S rRNA gene sequences analysis revealed that the strain chi1^T^ belonged to the genus *Marinobacter* in the family *Phyllobacteriaceae*, while the strain chi5^T^ belonged to the genus *Wenyingzhuangia* in the family *Flavobacteriaceae*. The completed 16S rRNA gene sequences of the novel strains were registered in EzBioCloud to confirm their similarity.

The strain chi1^T^ showed the highest similarity to *M. nitratireducens* AK21^T^ (97.5%), followed by *M*. *pelagius* HS225^T^ (97.3%), *M*. *mobilis* CN46^T^ (97.3%), and *M*. *salinexigens* ZYF650^T^ (97.3%), while the strain chi5^T^ showed the highest similarity to *W. marina* DSM 100572^T^ (98.8%), followed by *W*. *fucanilytica* CZ1127^T^ (97.2%), *W*. *heitensis* H-MN17^T^ (97.2%), *W*. *aestuarii* LC052784^T^ (95.8%), and *W*. *gracilariae* N5DB13-4^T^ (95.7%). Based on the 16S rRNA gene sequences, phylogenetic trees were constructed using the ML, NJ, and MP methods.

The phylogenetic analysis revealed that the two strains (chi1^T^ and chi5^T^) each clustered into a clade with their closely related *M. nitratireducens* AK21^T^ and *W. marina* DSM 100572^T^, respectively (Figure S1). However, low bootstrap values and relatively long branches between the closest relatives in the phylogenetic trees indicate that these strains could be classified as novel species within the genera *Marinobacter* and *Wenyingzhuangia*, respectively. Present tendencies imply that relying solely on lineages and similarity results based on the 16S rRNA gene is not dependable for identifying the close phylogenetic relatives of novel lineages. Consequently, this suggests that genome-wide comparisons should be emphasized as the primary criterion [57].

### 3.3. Genomic Features

Self-mapping and BUSCO analysis revealed that the strains chi1^T^ and chi5^T^ exhibited high-quality genomic integrity. The strain chi1^T^ showed 99.99% mapped reads and the strain chi5^T^ had 99.87%, both achieving 100% mapping coverage and complete BUSCO scores. The assembled draft genome sequence of the strain chi1^T^ was 3,572,209 bp in size, with a contig N50 value of 1,388,190 bp. It contained 6 contigs; 6 rRNAs; 46 tRNAs; and 3286 total genes, including 3230 protein-coding sequences (CDSs); and the genomic DNA G + C content was 57.2%. The assembled draft genome sequence of the strain chi5^T^ was 3,353,634 bp in size, with a contig N50 value of 383,849 bp. It contained 34 contigs; 6 rRNAs; 45 tRNAs; and 2909 total genes, including 2854 CDSs; and the genomic DNA G + C content was 31.5%. The detailed profiles of the genome of the novel strains and closely related strains are shown in Table S1. Analysis by the NCBI database revealed that chi1^T^’s genes included the flagellar and motility genes *Fli*L and *Mot*A/*Tol*Q/*Exb*B. The details of genes associated with motility in chi1^T^ are presented in Table S2. The analysis using CheckM indicated that the novel strains chi1^T^ and chi5^T^ had completenesses of 99.6% and 97.9%, respectively. The distribution of functional categories in the strains chi1^T^ and chi5^T^ annotated using the RAST server is presented in Table S3. Both strains contained amino acids and derivatives, carbohydrates, and protein metabolism as the most abundant RAST subsystems. Next, the antiSMASH analysis of the genome of strain chi1^T^ revealed eight gene clusters: one ectoine, one redox cofactor, one RiPP-like, one T1PKS, one NRPS-like, one NRPS, and two beta-lactone gene clusters. Two gene clusters were identified in the strain chi5^T^: one RiPP-like and one terpene gene cluster. The antiSMASH results of the novel strains are detailed in Table S4. A total of 2288 OCs were shared between the strains *M*. *suaedae* chi1^T^, *M*. *nitratireducens* AK21^T^, and *M*. *salinexigens* ZYF650^T^. Of these, 148 OCs were shared between the strains *M*. *suaedae* chi1^T^ and *M*. *nitratireducens* AK21^T^, while 77 OCs were shared between the strains *M*. *suaedae* chi1^T^ and *M*. *salinexigens* ZYF650^T^. A total of 1878 OCs were shared between the strains *W*. *gilva* chi5^T^, *W*. *fucanilytica* CZ1127^T^, and *W*. *marina* DSM 100572^T^. Of these, 75 OCs were shared between the strains *W*. *gilva* chi5^T^ and *W*. *fucanilytica* CZ1127^T^, while 65 OCs were shared between the strains *W*. *gilva* chi5^T^ and *W*. *marina* DSM 100572^T^.

**Figure 1:**
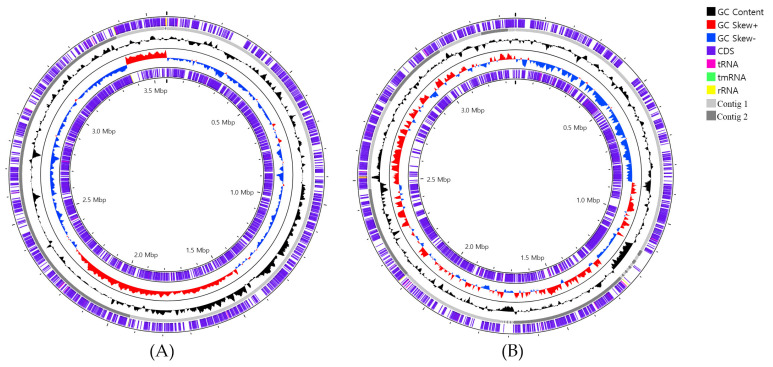
Genomic circular feature maps of strains (**A**) chi1^T^ and (**B**) chi5^T^.

In addition, we further demonstrated that the strains chi1^T^ and chi5^T^ had 22 and 20 independent gene clusters, respectively, compared with the reference strains. UpSet plots of the detailed OCs in the genera *Marinobacter* and *Wenyingzhuangia* are presented in Figure S2. Genomic circular feature maps of the two novel strains were constructed using the CGView server, as shown in Figure 1.

The ANI and dDDH values between the strain chi1^T^ and some *Marinobacter* species ranged from 74.4% to 76.5% and 19.0% to 20.0%, respectively. Moreover, the ANI and dDDH values between the strain chi5^T^ and some *Wenyingzhuangia* species ranged from 79.6% to 88.9% and 22.1% to 36.6%, respectively.

The analysis of ANI values across 8 billion genome pairs, including the novel strain chi5^T^, underlines a clear genetic discontinuity, with 99.8% adhering to intra-species ANI values above 95% and inter-species values below 83% [58]. The strain chi5^T^ showed ANI values of 84.6% with *W*. *fucanilytica* and 88.9% with *W*. *marina*, indicating genetic distinctness while sharing a close phylogenetic relationship.

**Figure 2:**
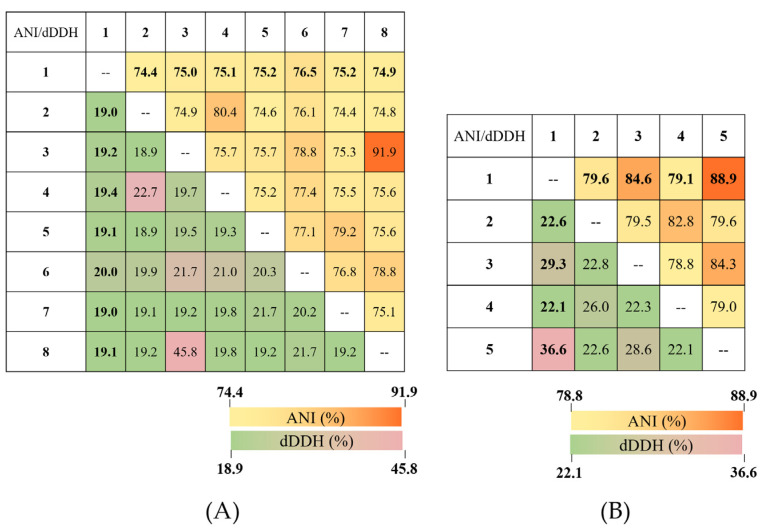
Heat maps of digital DNA–DNA hybridization (dDDH) and average nucleotide identity (ANI) values (**A**) between strain chi1^T^ and species belonging to the genus *Marinobacter* and (**B**) between strain chi5^T^ and species belonging to the genus *Wenyingzhuangia*. (**A**) Strains: 1, *M*. *suaedae* chi1^T^; 2, *M*. *daepoensis* DSM 16072^T^; 3, *M*. *koreensis* DD-M3^T^; 4, *M*. *nauticus* ATCC 49840^T^; 5, *M*. *nitratireducens* AK21^T^; 6, *M*. *pelagius* HS225^T^; 7, *M*. *salinexigens* ZYF650^T^; and 8, *M*. *santoriniensis* NKSG1^T^. (**B**) Strains: 1, *W*. *gilva* chi5^T^; 2, *W*. *aestuarii* DSM 105044^T^; 3, *W*. *fucanilytica* CZ1127^T^; 4, *W*. *heitensis* DSM 101599^T^; and 5, *W*. *marina* DSM 100572^T^.

Detailed ANI and dDDH heat maps for the genera *Marinobacter* and *Wenyingzhuangia* are shown in Figure 2.

The two species chi1^T^ and chi5^T^ did not fall within the boundaries of generally accepted stable values based on the recommended thresholds of 95–96% for ANI values [59] and 70% for dDDH values [60]. These values indicate that the two novel strains were clearly separated from known taxa at the same species level. In addition, the ANI and dDDH values between chi5^T^ and *W*. *marina* DSM 100572^T^ were relatively high but not acceptable for the recommended thresholds.

**Figure 3:**
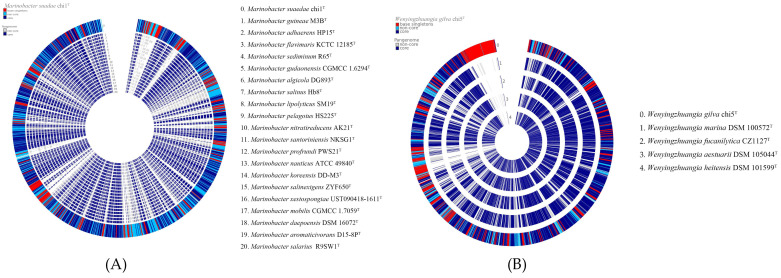
Pangenomes of strains (**A**) chi1^T^ and (**B**) chi5^T^. Pangenomes were created using Build Pangenome with OrthoMCL version 2.0. Red indicates base singletons, dark blue indicates core genes, and sky blue indicates noncore genes. Moreover, noncore genes in the pangenome of the reference strain are indicated in gray.

The strain chi1^T^ contained 52 ORFs that encoded CAZymes (7 AAs, 7 CEs, 8 GHs, and 30 GTs), while the strain chi5^T^ contained 218 ORFs encoding CAZymes (7 AAs, 22 CEs, 122 GHs, 52 GTs, and 15 PLs). The details regarding the CAZymes in the novel strains and reference strains are presented in Table S5. A comparison of the pangenomes of the novel strains chi1^T^ and chi5^T^ with those of the closely related species revealed the distinct genetic makeup and unique status of the two novel strains (Figure 3). The pangenomic analysis revealed that the genome of the novel strain chi1^T^ contained a total of 3230 genes, including 2873 homologous genes, 357 singleton genes, and 2804 homologous families, while that of the novel strain chi5^T^ contained a total of 2855 genes, including 2487 homologous genes, 368 singleton genes, and 2411 homologous families. The pangenomic datasets of the novel strains and closely related species are presented in Table S6.

**Figure 4:**
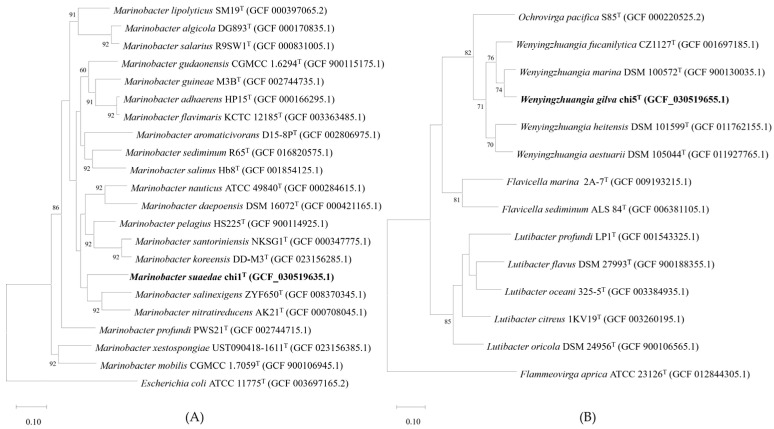
The phylogenomic trees were constructed based on the UBCG relationship between chi1^T^, chi5^T^, and their closely related strains. Numbers at nodes designate bootstrap values, expressed as a percentage of 100 replicates, with values below 60% not shown. Based on the phylogenomic tree construction results, the strain chi1^T^ (**A**) and the strain chi5^T^ (**B**) were classified as being in the same clade as the genera *Marinobacter* and *Wenyingzhuangia*, respectively.

In the UBCG phylogenomic tree constructed based on whole-genome shotgun sequences, the strain chi1^T^ was closely clustered with *M*. *salinexigens* ZYF650^T^ and *M*. *nitratireducens* AK21^T^, while the strain chi5^T^ was closely clustered with *W*. *fucanilytica* CZ1127^T^ and *W*. *marina* DSM 100572^T^ (Figure 4).

Therefore, the strains (*M*. *salinexigens* ZYF650^T^, *M*. *nitratireducens* AK21^T^, *W*. *fucanilytica* CZ1127^T^, and *W*. *marina* DSM 100572^T^) that formed independent clusters were determined to be the reference strains for the taxonomic studies.

### 3.4. Physiology and Chemotaxonomy

The strain chi1^T^ only grew well on marine agar, while the strain chi5^T^ grew well on LB agar, R2A agar, and marine agar and grew weakly on NA. The strains chi1^T^ and chi5^T^ grew at temperatures of 20–45 °C (optimum, 35 °C) and 20–38 °C (optimum, 30 °C), respectively, and at pH values of 6.0–9.0 (optimum, 7.0) and 6.0–8.0 (optimum, 7.0), respectively. Moreover, the strain chi1^T^ could grow at NaCl concentrations of 0–15% (*w*/*v*; optimum, 2%), while chi5^T^ could grow at NaCl concentrations of 0–5% (*w*/*v*; optimum, 2%). The novel strains were positive for the catalase and oxidase activities, producing oxygen bubbles in the presence of a 3% (*v*/*v*) aqueous hydrogen peroxide solution and changing the cell color to dark blue, respectively. The cells of the novel strains were Gram-negative and appeared rod-shaped under a TEM (Figure S3). The strain chi1^T^ did not produce flagella when observed by TEM (Figure S4). The hydrolytic reactions of the novel strains were all negative. Comparisons of the biochemical and phenotypic characteristics of the strains are presented in Table 1.

The major fatty acids of the strain chi1^T^ were C_12:0_, C_16:0_, and summed feature 3 (C_16:1_ *ω*6*c* and/or C_16:1_ *ω*7*c*), while those of the strain chi5^T^ were iso-C_15:0_ 3OH, iso-C_17:0_ 3OH, and iso-C_15:0_. Details regarding the fatty acids are presented in Table S7.

The major fatty acid profiles of the strains chi1^T^ and chi5^T^ exhibited notable variances when compared with the reference strains. Specifically, the strain chi1^T^ contained a higher amount of summed feature 3 (C_16:1_ *ω*6c and/or C_16:1_ *ω*7c) and a lower amount of C_18:1_ *ω*9*c*. Conversely, the strain chi5^T^ contained a higher amount of Iso-C_15:0_. These significant differences in fatty acids allowed us to demonstrate that the strains chi1^T^ and chi5^T^ were clearly distinct from their respective similar species.

The polar lipids of the strain chi1^T^ consisted of one PL, one phosphatidylglycerol (PG), one diphosphatidylglycerol (DPG), one aminoglycolipid (AGL), and one phosphatidylethanolamine (PE). The major polar lipids were PE, PG, and DPG. The strain chi5^T^ contained one PE, four GLs, one AL, one AGL, and one unidentified lipid (UL). The major polar lipid was PE. Details regarding the polar lipids are presented in Figure S5 and Table S8.

The strain chi1^T^ contained the same major polar lipids, one DPG, and one PG as the reference strains [61]. The strain chi5^T^ shared one PE with the reference strains and additionally contained four unique GLs [21,23]. The presence of the same major polar lipids demonstrated that the two novel strains chi1^T^ and chi5^T^ were closely related to their respective species.

### 3.5. Description of *Marinobacter Suadae* sp. nov.

#### *Marinobacter suadae* (su.ae’dae. L. gen. n. suaedae, from *Suaeda*, the Plant)

The cells of the strain chi1^T^ were Gram-negative, aerobic, nonmotile, and approximately 0.52–0.57 μm wide and 1.67–2.62 μm long. The colonies that were grown on marine agar plates were circular, light ochre, and smooth. The catalase and oxidase reactions were negative. The cells only grew well on marine agar. The strain grew at temperatures of 20–45 °C (optimum, 35 °C), at pH values of 6.0–9.0 (optimum, 7.0), and in the presence of 0–15% NaCl (*w*/*v*; optimum, 2%). In the API 20NE assay, the strain was only positive for the assimilation of esculin. The other reactions were negative. In the API 50CH assay, the strain exhibited acid production from D-galactose, methyl α-D-mannopyranoside, cellobiose, melezitose, and 5-ketogluconate but not from D-lyxose, N-acetylglucosamine, DL-arabitol, starch, turanose, melibiose, L-rhamnose, lactose, amygdalin, D-fructose, D-mannose, erythritol, potassium gluconate, 2-ketogluconate, sucrose and maltose, xylitol or DL-xylose, inulin, trehalose, L-sorbose, D-tagatose, D-sorbitol, D-mannitol, gentiobiose, D-glucose, dulcitol, DL-arabinose, raffinose, esculin, salicin, arbutin, inositol, D-ribose, methyl β-D-xylopyranoside, DL-fucose, glycerol, D-adonitol, and glycogen. The major fatty acids (>10%) were C_12:0_, C_16:0_, and summed feature 3 (C_16:1_ *ω*6*c* and/or C_16:1_ *ω*7*c*). The major polar lipids were PE, PG, and DPG.

The strain chi1^T^ (= KACC 23259^T^ = TBRC 17652^T^) was isolated from the root of *S. japonica* Makino in Seongmodo, South Korea. The DNA G + C content of the type strain was 57.2 mol%.

The GenBank/EMBL/DDBJ/PIR accession numbers for the 16S rRNA gene sequences and the whole-genome shotgun sequences of *M. suadae* chi1^T^ are OQ781157 and JAUMIS000000000, respectively.

### 3.6. Description of *Wenyingzhuangia gilva* sp. nov.

#### *Wenyingzhuangia gilva* (gil’va. L. fem. adj. *gilva*, Yellow)

The cells of the strain chi5^T^ were Gram-negative, aerobic, nonmotile, and approximately 0.28–0.33 μm wide and 1.14–1.33 μm long. The colonies that were grown on LB agar were circular, yellow, and convex. Moreover, the colonies that were grown on R2A agar were spread, pale yellow, and watery. The strain grew weakly on NA and marine agar but did not grow on TSA. The strain grew at temperatures of 20–38 °C (optimum, 30 °C), at pH values of 6.0–8.0 (optimum, 7.0), and in the presence of 0–5% NaCl (*w*/*v*; optimum, 2%). In the API 20NE assay, the strain was only positive for the assimilation of esculin. Other reactions were negative. In the API 50CH assay, the strain exhibited acid production from cellobiose, maltose, lactose, esculin, L-arabinose, and 5-ketogluconate but not from D-galactose, methyl α-D-mannopyranoside, melezitose, D-lyxose, N-acetylglucosamine, DL-arabitol, starch, turanose, melibiose, L-rhamnose, amygdalin, D-fructose, D-mannose, erythritol, potassium gluconate, 2-ketogluconate, sucrose, xylitol or DL-xylose, inulin, trehalose, L-sorbose, D-tagatose, D-sorbitol, D-mannitol, gentiobiose, D-glucose, dulcitol, D-arabinose, raffinose, salicin, arbutin, inositol, D-ribose, methyl β-D-xylopyranoside, DL-fucose, glycerol, D-adonitol, and glycogen. The major fatty acids (>10%) were iso-C_15:0_ 3OH, iso-C_17:0_ 3OH, and iso-C_15:0_. The major polar lipid was PE.

Strain chi5^T^ (= KACC 23262^T^ = TBRC 17900^T^) was isolated from the root of *S. japonica* Makino in Seongmodo, South Korea. The DNA G + C content of the type strain was 31.5 mol%.

The GenBank/EMBL/DDBJ/PIR accession numbers for the 16S rRNA gene sequences and the whole-genome shotgun sequences of *W. gilva* chi5^T^ are OQ781158 and JAUMIT000000000, respectively.

## 4. Conclusions

Phylogenetic, genomic features comparative genomic analyses, and chemotaxonomic characteristics indicated that chi1^T^ and chi5^T^ represent novel species placed in the genera *Marinobacter* and *Wenyingzhuangia*, respectively.

The composition of the major fatty acids in the reference strains and the two novel strains chi1^T^ and chi5^T^ were similar, but some differences could be identified. Furthermore, the presence of the same major polar lipids indicated that they were similar species.

In the case of the strain chi1^T^ in particular, the potential for PAH degradation exists due to the association of marine habitats with *Marinobacter* species that degrade PAHs. In addition, the presence of flagellar and motility genes suggests that the potential for expression exists. These two possibilities would be logical targets for future studies.

In summary, the novel strains chi1^T^ and chi5^T^ possess several features that clearly distinguish them from the genera *Marinobacter* and *Wenyingzhuangia*.

Therefore, we propose the names *Marinobacter suadae* sp. nov. for the strain chi1^T^ and *Wenyingzhuangia gilva* sp. nov. for the strain chi5^T^.

## Figures and Tables

**Table 1:** Differential characteristics between the novel strains chi1^T^ and chi5^T^ and reference strains in the genus *Marinobacter* (A) and *Wenyingzhuangia* (B).

(A)			
Characteristics	Species		
	1	2	3
Colony color	Cream	Cream	Yellow
Optimum temperature range (°C)	35	28–35	26–28
Temperature range (°C)	20–45	15–42	10–45
Optimum pH	7.0	7.0	7.0
pH range	6.0–9.0	6.0–10.0	6.0–9.5
NaCl concentration range (%, *w*/*v*)	0–15	0–6	0–14
Catalase	−	+	+
Oxidase	−	+	−
Hydrolysis of:			
Agar	−	−	−
Casein	−	−	−
Chitin	−	−	−
DNase	−	−	+
Tween 20	−	+	−
Tween 80	−	+	−
Gelatin	−	−	+
Starch	−	−	+
Assimilation of (API 20NE):			
Esculin	+	+	+
Acid production from:			
D-galactose	+	−	−
Methyl-α-D-mannopyranoside	+	−	−
Cellobiose	+	−	+
Melezitose	+	−	−
5-Ketogluconate	+	+	−
**(B)**			
**Characteristics**	**Species**		
	**1**	**2**	**3**
Colony color	Yellow	Yellow	Yellow
Optimum temperature range (°C)	30	25	25
Temperature range (°C)	20–40	15–37	15–30
Optimum pH	7.0	6.5–7.5	6.5–7.0
pH range	6.0–8.0	5.5–9.0	5.5–9.0
NaCl concentration range (%, *w*/*v*)	0–5	0–4	0–8
Catalase	+	+	+
Oxidase	+	+	+
Hydrolysis of:			
Agar	−	−	−
Casein	−	−	−
Tween 20	−	−	−
Tween 80	−	−	−
Gelatin	−	−	−
Starch	−	+	+
Assimilation of (API 20NE):			
Esculin	+	+	+
4-Nitrophenyl-β-D-galactopyranoside	+	−	−
Acid production from:			
L-arabinose	+	−	−
Esculin	+	+	+
Cellobiose	+	+	+
Maltose	+	−	+
Lactose	+	+	+
5-Ketogluconate	+	−	−

(A) Strains: 1, *M*. *suaedae* chi1; 2, *M*. *nitratireducens* AK21^T^; 3, *M*. *salinexigens* ZYF650^T^. +, positive; −, negative. (B) Strains: 1, *W*. *gilva* chi5^T^; 2, *W*. *fucanilytica* CZ1127^T^; 3, *W*. *marina* DSM 100572^T^. +, positive; −, negative.

## Data Availability

Data are contained within the article and Supplementary Materials.

## Supplementary Materials

The following supporting information can be downloaded from https://www.mdpi.com/article/10.3390/life14030296/s1—Figure S1: 16S rRNA gene analysis based on phylogenetic trees constructed using the ML, NJ, and MP algorithms. Figure S2: UpSet table showing the orthologous clusters of strain chi1T and their closely related reference strains (A) and strain chi5T and their closely related reference strains (B). Figure S3: Strains chi1T and chi5T were incubated at 30 °C for 3 days on marine agar and LB agar, respectively, and observed under a transmission electron microscope (JEM-1010; JEOL). Figure S4: Presence of flagella according to NaCl concentration (*w*/*v*) change in strain chi1T. Figure S5: Results of two-dimensional thin-layer chromatography (TLC) showing the polar lipid profiles of the two novel strains chi1T and chi5T. Table S1: Genomic features of strains chi1T and chi5T and reference strains belonging to the genera Marinobacter and Wenyingzhuangia, respectively. Table S2: Flagella and motility gene profiles of strain chi1T. Table S3: Secondary metabolite gene cluster profiles of strains chi1T and chi5T. Table S4: Subsystem features of strains chi1T and chi5T and reference strains belonging to the genera Marinobacter and Wenyingzhuangia, respectively, as assessed using the RAST server. Table S5: Number of ORFs in the novel strains chi1T and chi5T compared with those in related species belonging to the genera Marinobacter and Wenyingzhuangia, respectively. Table S6: Results of pangenomic analysis of the novel strains chi1T and chi5T and reference strains belonging to the genera Marinobacter and Wenyingzhuangia, respectively, using Build Pangenome with OrthoMCL version 2.0. Table S7: Cellular fatty acid profiles of strains chi1T and chi5T and closely related reference strains. Table S8: Polar lipid profiles of strains chi1T and chi5T and closely related reference strains.
