# Supplementary material for: Draft Genome Sequence Analyses of Two Novel Marinobacter suadae sp. nov. and Wenyingzhuangia gilva sp. nov. Isolated from the Root of Suaeda japonica Makino
Source: Life (Basel). 2024 Feb 22;14(3):296. doi: 10.3390/life14030296 (PMC10971456; doi:10.3390/life14030296)
Supplement: Supplementary file 1 [file life-14-00296-s001.zip › life-2820205-supplementary.pdf]

Supplementary Materials

Draft Genome Sequence Analyses of Two Novel *Marinobacter suadae* sp. nov. and *Wenyingzhuangia gilva* sp. nov., Isolated from the Root of *Suaeda japonica* Makino

Department of Life Science, Dongguk University-Seoul, Goyang 10326, Republic of Korea; eksvnd97@dgu.ac.kr (S.P.); duckling91@dgu.ac.kr (I.K.); lucky_salman@dongguk.edu (G.C.); joh2395@dongguk.edu (Y.J.); woohj999@dongguk.edu (H.W.)

***Corresponding author:**

Taegun Seo

Tel: +82-31-961-5135

Fax: +82-31-961-5348

E-mail: tseo@dongguk.edu

Fig. S1. 16S rRNA gene analysis based on phylogenetic trees constructed using the ML, NJ, and MP algorithms.

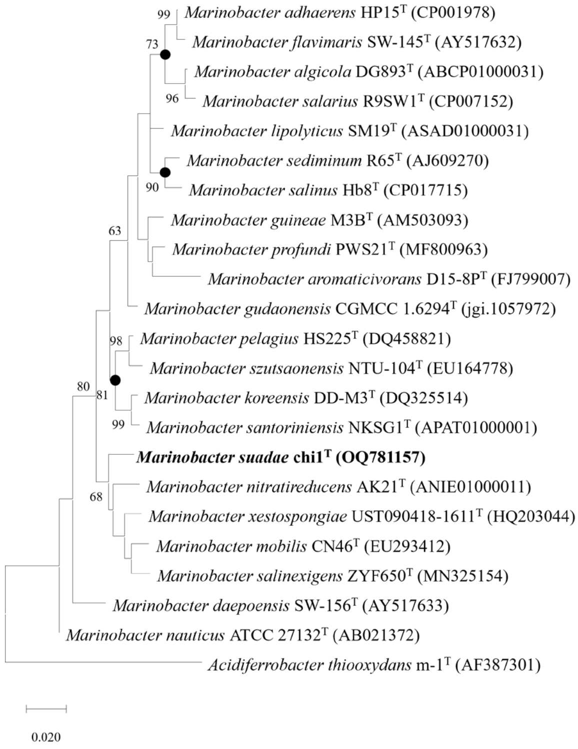

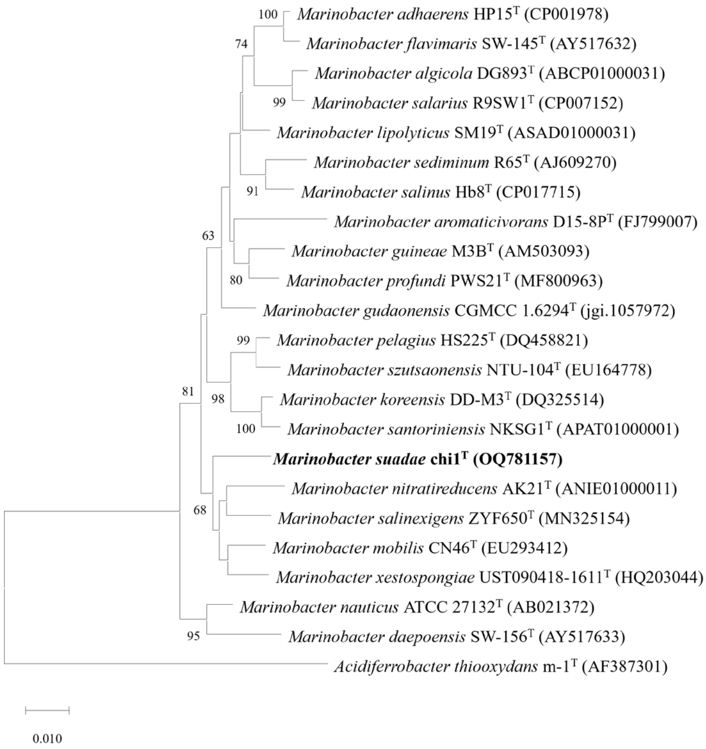

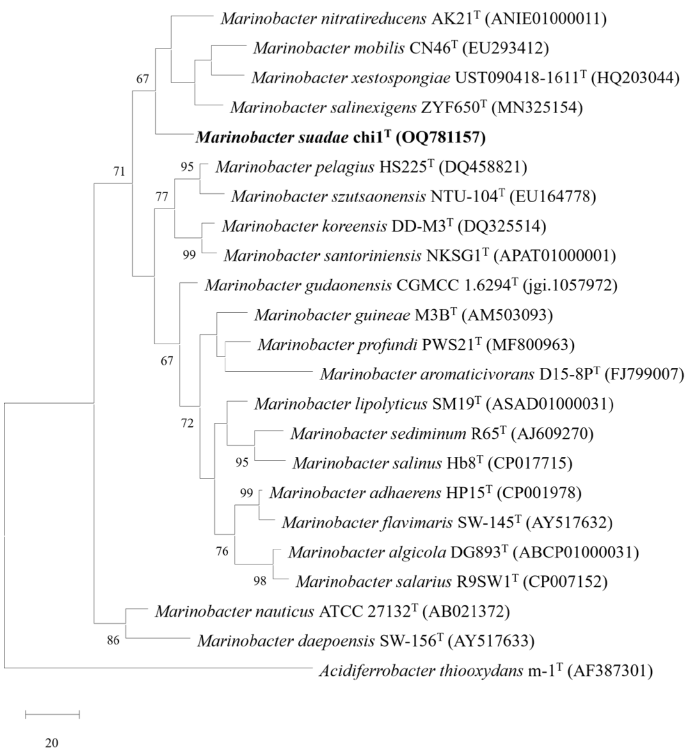

(A) Strain chi1^{T} within the genus *Marinobacter*.

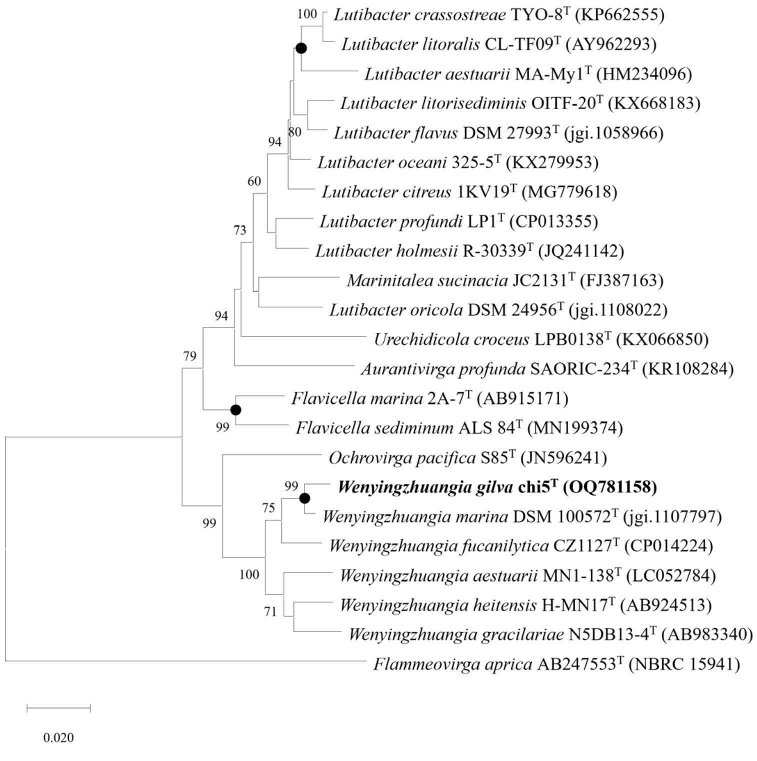

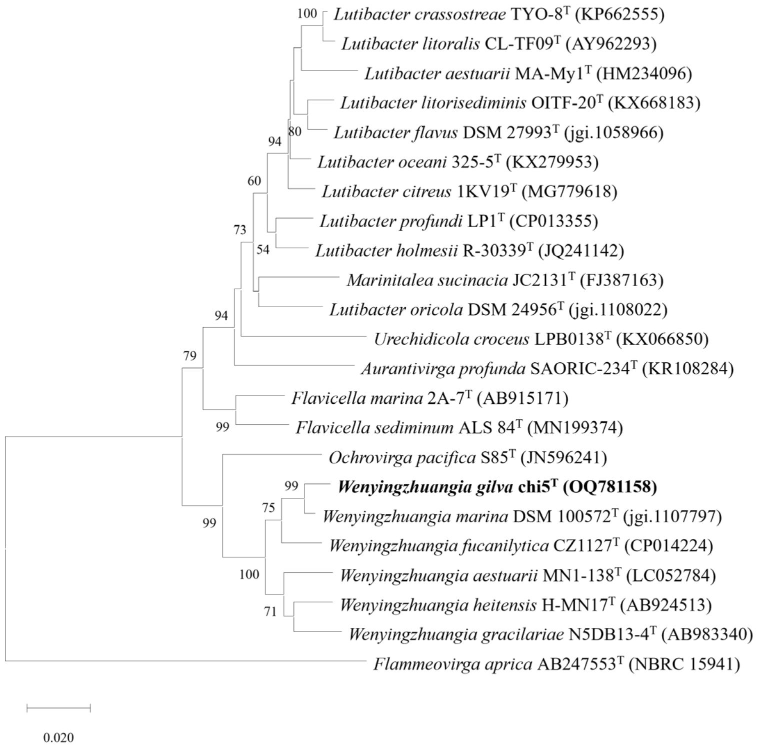

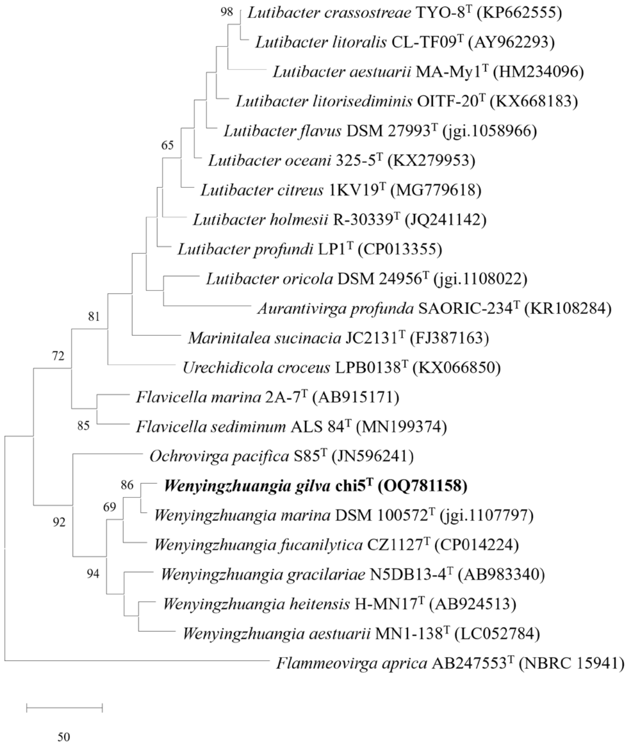

(B) Strain chi5^{T} within the genus *Wenyingzhuangia.*

The bootstrap was set as a percentage of 1,000 replicates (above 60%). The outgroups used were *Acidiferrobacter thiooxydan* m-1^{T} (AF387301) and *Flammeovirga aprica* AB247553^{T} (NBRC 15941).

Fig. S2. UpSet table showing the orthologous clusters of strain chi1^{T} and their closely related reference strains (A), while strain chi5^{T} and their closely related reference strains (B).

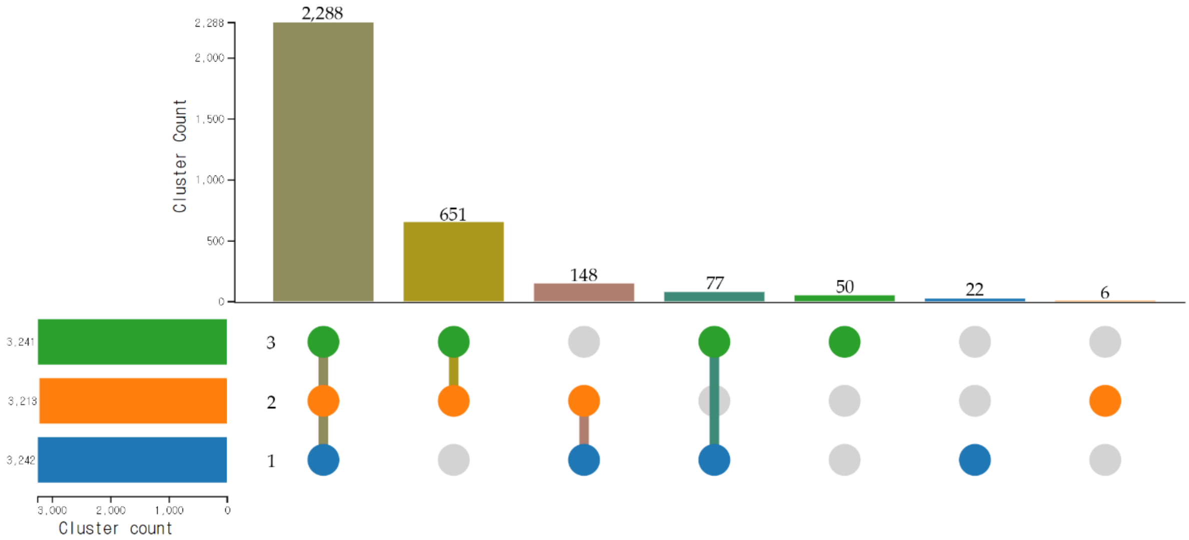

(A) *Marinobacter suadae* sp. nov.

Strains: 1, *M. suaedae* chi1; 2, *M. nitratireducens* AK21^{T}; 3, *M. salinexigens* ZYF650^{T}.

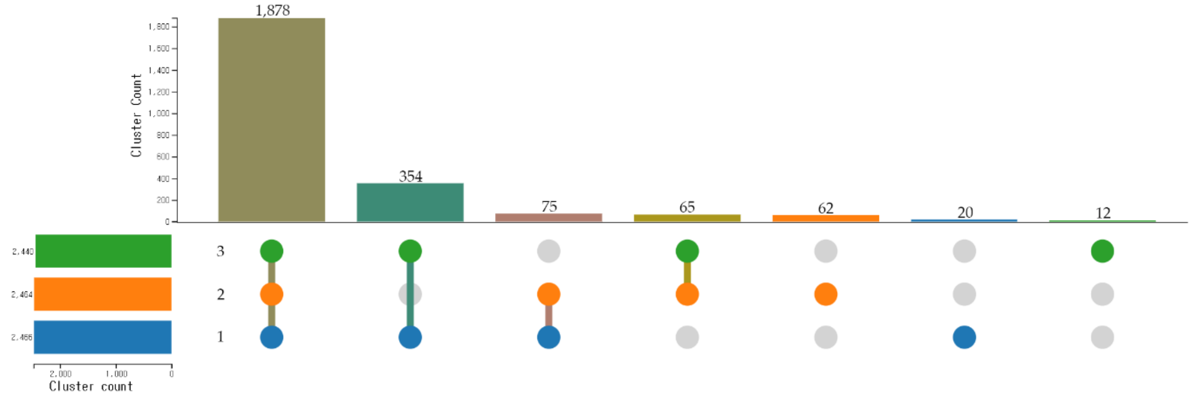

(B) *Wenyingzhuangia gilva* sp. nov.

Strains: 1, *W. gilva* chi5^{T}; 2, *W. fucanilytica* CZ1127^{T}; 3, *W. marina* DSM 100572^{T}.

Fig. S3. Strains chi1^T and chi5^T were incubated at 30°C for 3 days on marine agar and LB agar, respectively, and observed under a transmission electron microscope (JEM-1010; JEOL).

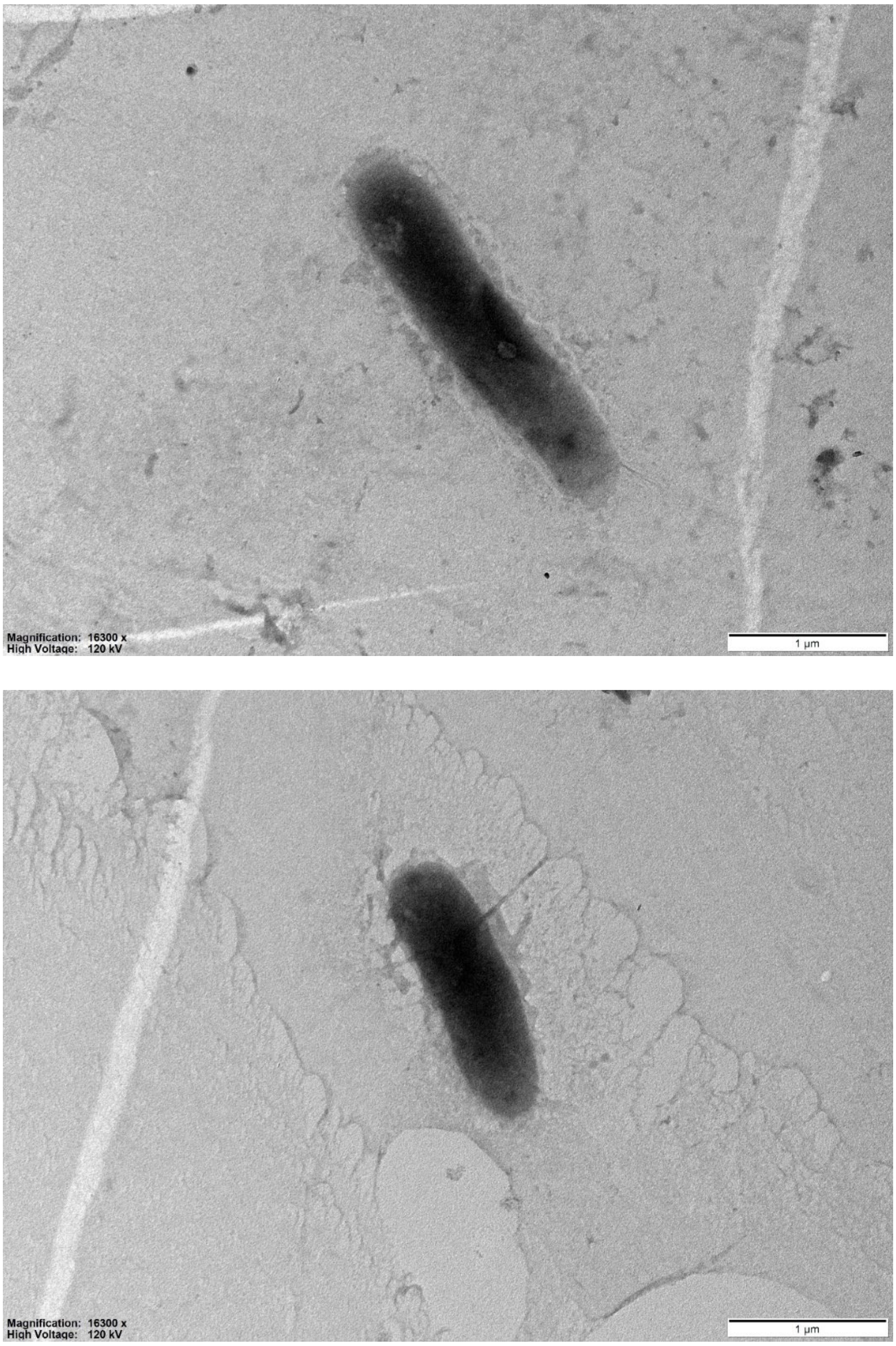

(A) *Marinobacter suadae* sp. nov.

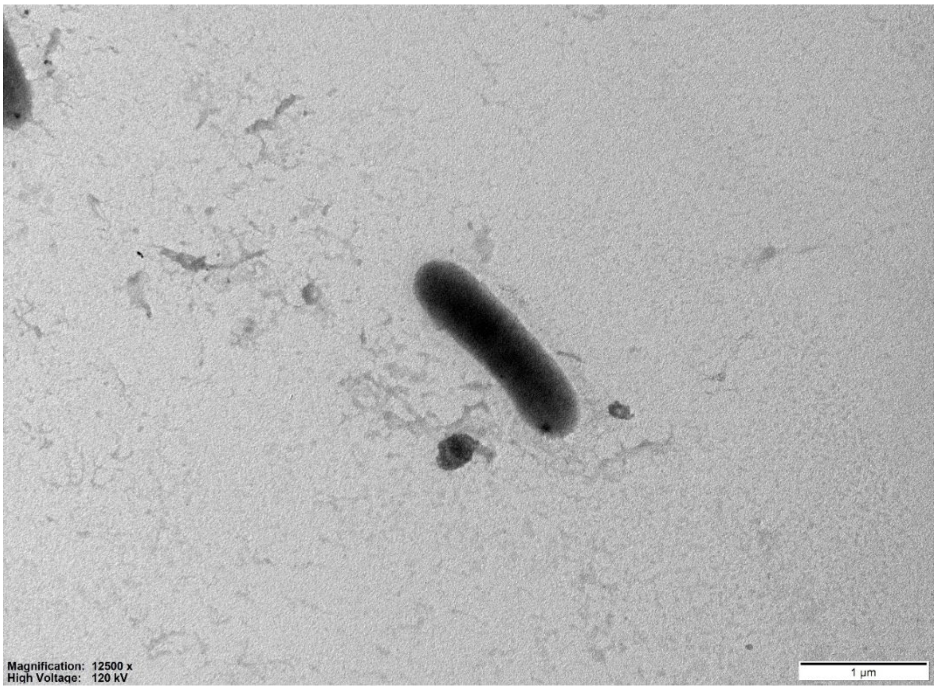

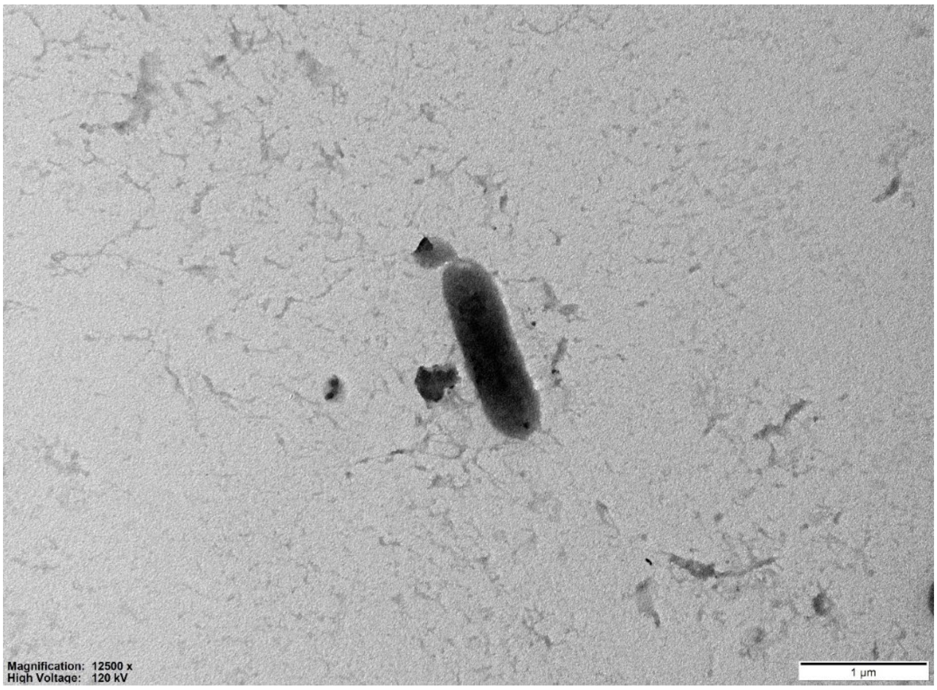

(B) *Wenyingzhuangia gilva* sp. nov.

Fig. S4. Presence of flagella according to NaCl concentration (w/v) change in strain chi1ᵀ.

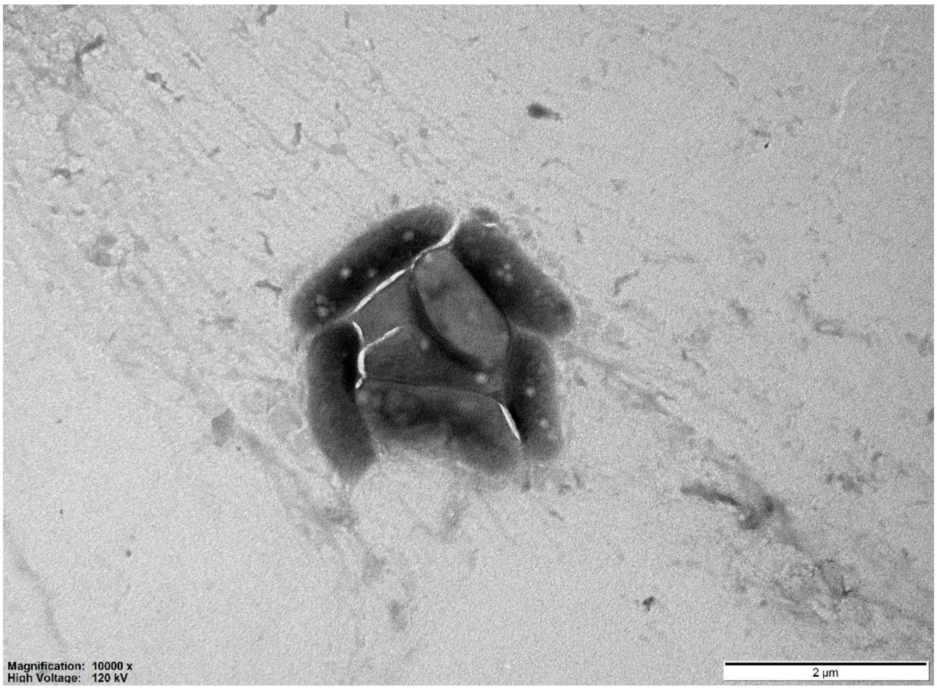

(A) *Marinobacter suadae* sp. nov. at a NaCl concentration of 3.5%.

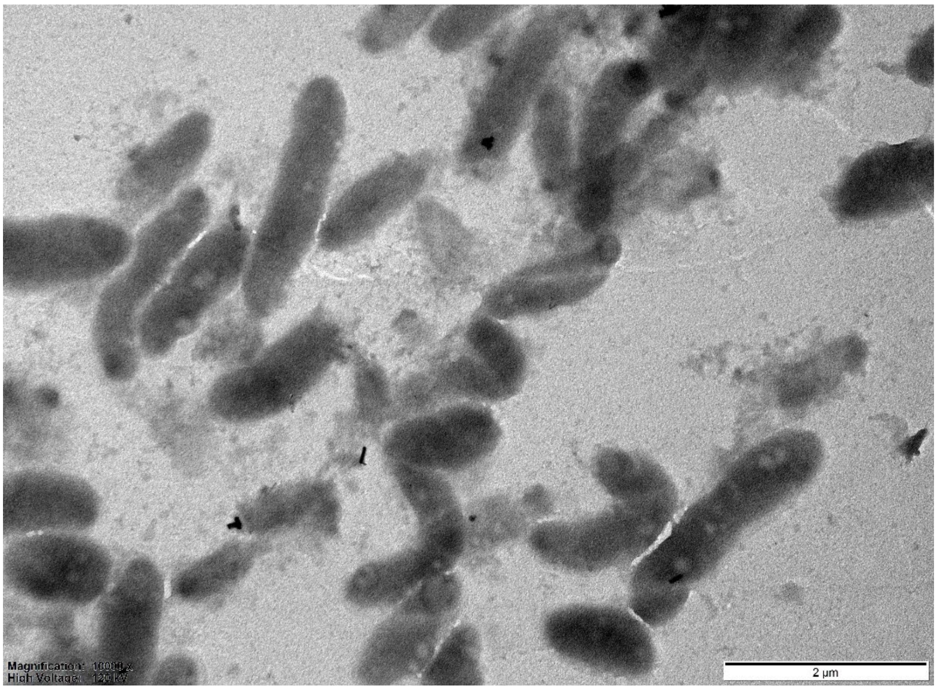

(B) *Marinobacter suadae* sp. nov. at a NaCl concentration of 9.0%.

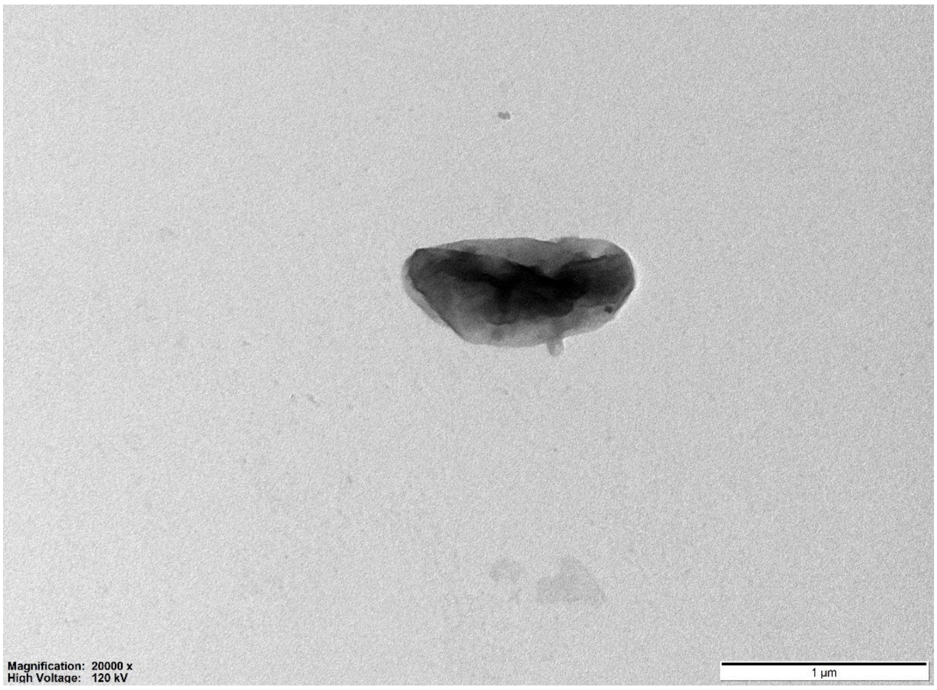

(C) *Marinobacter suadae* sp. nov. at a NaCl concentration of 15.0%.

Fig. S5. Results of two-dimensional thin-layer chromatography (TLC) showing the polar lipids profiles of the two novel strains chi1^T and chi5^T. A chloroform/methanol/water mixture (65:25:4, v/v/v) and a chloroform/acetic acid/methanol/water mixture (80:15:12:4, v/v/v/v) were used for the first and second directions, respectively.

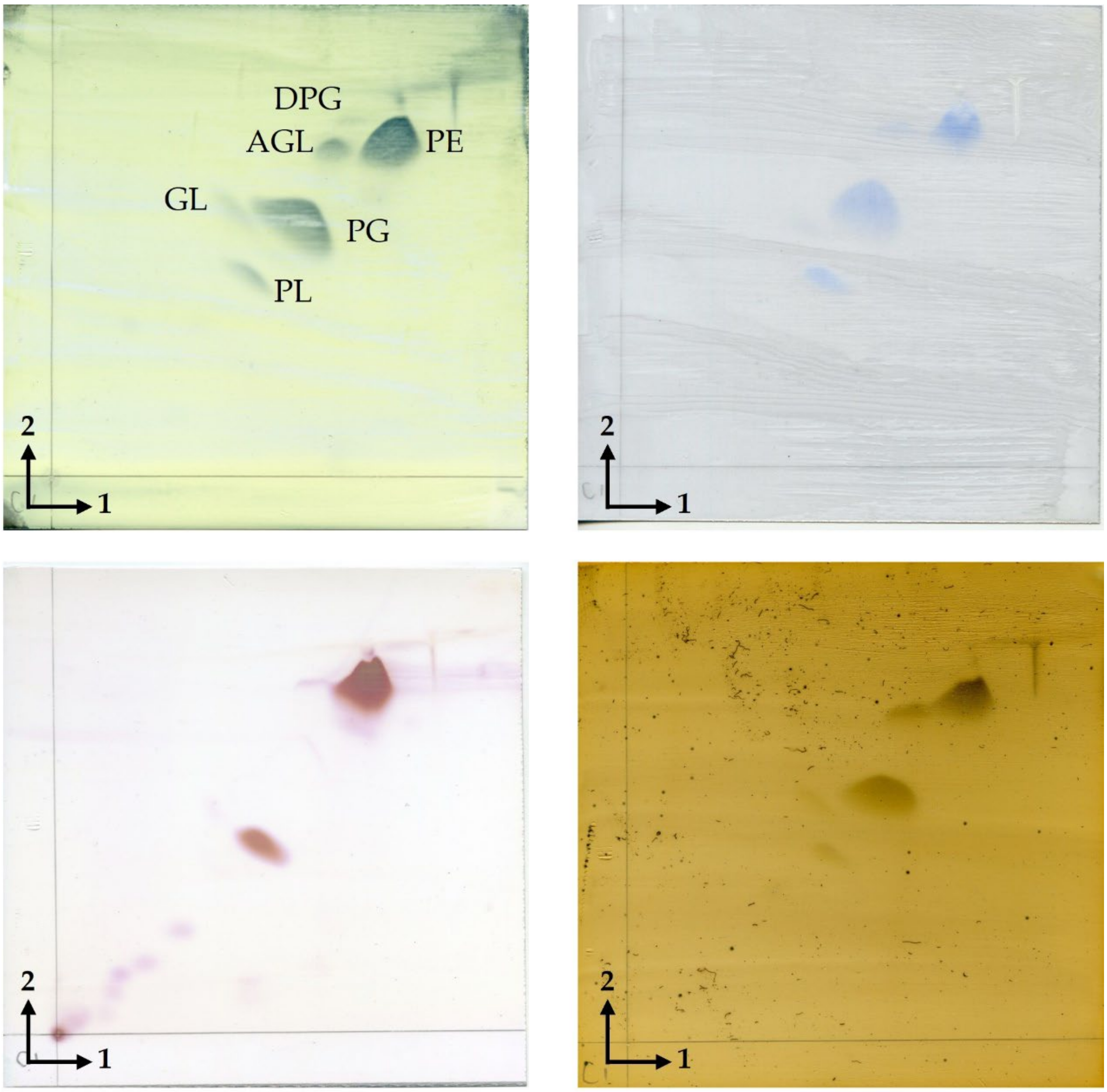

(A) *Marinobacter suadae* sp. nov.

one phospholipid (PL), one phosphatidylglycerol (PG), one diphosphatidylglycerol (DPG), one aminoglycolipid (AGL), and one phosphatidylethanolamine (PE).

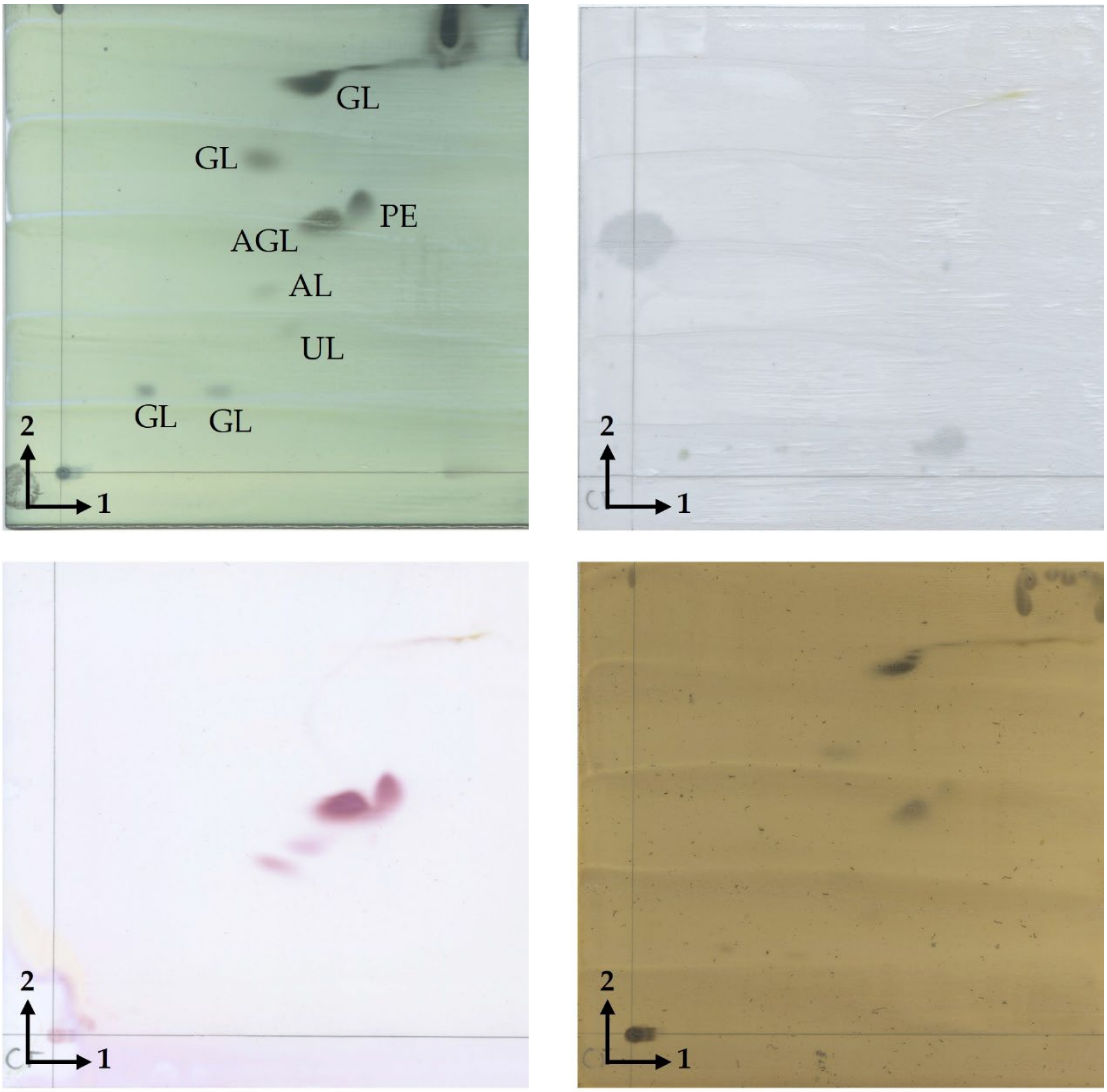

(B) *Wenyingzhuangia gilva* sp. nov.

one phosphatidylethanolamine (PE), four glycolipids (GLs), one aminolipid (AL), one aminoglycolipid (AGL), and one unidentifed lipid (UL).

Table S1. Genomic features of strains chi1^T and chi5^T and reference strains belonging to the genera *Marinobacter* and *Wenyingzhuangia*, respectively.

(A) Genomic features of strain chi1^T and reference strains.

Characteristics	Species		
	1	2	3
Genome length (bp)	3,572,209	3,890,033	4,240,922
Contigs	6	22	149
Contig N50 bp	1,388,190	552,815	527,644
Number of rRNAs	6	3	7
Number of tRNAs	46	45	50
Total gene count	3,286	3,543	3,915
Total protein count	3,230	3,491	3,854
DNA G + C content (%)	57.2	56.7	55.6

Strains: 1, *M. suaedae* chi1^T; 2, *M. nitratireducens* AK21^T; 3, *M. salinexigens* ZYF650^T.

(B) Genomic features of strain chi5^T and reference strains.

Characteristics	Species				
	1	2	3	4	5
Genome length (bp)	3,353,634	3,526,759	3,427,057	3,668,833	3,242,070
Contigs	34	6	N/D	29	12
Contig N50 bp	383,849	201,914	N/D	1,935,568	707,963
Number of rRNAs	6	4	12	3	3
Number of tRNAs	45	45	52	45	45
Total gene count	2,909	3,045	2,871	3,123	2,810
Total protein count	2,854	2,961	2,792	3,033	2,737
DNA G+C content (%)	31.5	31.8	31.6	31.4	31.2

Strains: 1, *W. gilva* chi5^T; 2, *W. aestuarii* DSM 105044^T; 3, *W. fucanilytica* CZ1127^T; 4, *W. heitensis* DSM 101599^T; 5, *W. marina* DSM 100572^T. N/D, no data.

Table S2. Flagella and motility gene profiles of strain chi1^T.

Gene	Accession	Length (aa)
*Fli*L	WP_302909520	51
*Mot*A/*Tol*Q/*Exb*B	WP_302909899	460

Table S3. Secondary metabolite gene cluster profiles of strains chi1^T and chi5^T.

(A) Gene cluster profiles of strain chi1^T.

Region	Type	From	To	Most similar known cluster	Similarity (%)
1.1	Ectoine	1,503,503	1,513,898	-	-
1.2	Redox-cofactor	1,747,656	1,769,819	Lankacidin C	13
2.1	RiPP-like	287,731	299,923	-	-
2.2	NRPS-like, T1PKS, NRPS	605,619	658,653	-	-
2.3	Betalactone	702,342	726,527	-	-
2.4	Betalactone	1,091,039	1,122,812	Plipastatin	15

(B) Gene cluster profiles of strain chi5^T.

Region	Type	From	To	Most similar known cluster	Similarity (%)
1.1	Ripp-like	529,970	540,800	-	-
6.1	Terpene	154,597	175,433	Carotenoid	28

Table S4. Subsystem features of strains chi1^T and chi5^T and reference strains belonging to the genera *Marinobacter and Wenyingzhuangia*, respectively, as assessed using the RAST server.

(A) Subsystem features of strain chi1^T and reference strains.

Subsystem category distribution	Strains		
	1	2	3
Amino acids and derivatives	285	290	272
Carbohydrates	149	211	176
Cell division and cell cycle	0	0	0
Cell wall and capsule	22	28	26
Cofactors, vitamins, prosthetic groups, and pigments	124	169	155
DNA metabolism	59	73	69
Dormancy and sporulation	4	3	3
Fatty acids, lipids, and isoprenoids	89	103	93
Iron acquisition and metabolism	3	7	8
Membrane transport	100	101	93
Metabolism of aromatic compounds	4	58	42
Miscellaneous	15	13	10
Motility and chemotaxis	0	76	32
Nitrogen metabolism	14	53	50
Nucleosides and nucleotides	73	74	59
Phages, prophages, transposable elements, and plasmids	2	7	5
Phosphorus metabolism	24	21	23
Photosynthesis	0	0	0
Potassium metabolism	9	8	7
Protein metabolism	198	204	197
Regulation and cell signaling	14	31	20
Respiration	60	80	65
RNA metabolism	51	54	52
Secondary metabolism	4	4	4
Stress response	56	64	70
Sulfur metabolism	9	16	6
Virulence, disease, and defense	31	53	40

Strains: 1, *M. suaedae* chi1^T; 2, *M. nitratireducens* AK21^T; 3, *M. salinexigens* ZYF650^T.

(B) Subsystem features of strain chi5^{T} and reference strains

Subsystem category distribution	Strains		
	1	2	3
Amino acids and derivatives	151	230	237
Carbohydrates	114	201	271
Cell division and cell cycle	3	26	27
Cell wall and capsule	26	65	68
Cofactors, vitamins, prosthetic groups, and pigments	127	145	143
DNA metabolism	48	67	74
Dormancy and sporulation	2	3	4
Fatty acids, lipids, and isoprenoids	18	43	56
Iron acquisition and metabolism	0	3	3
Membrane transport	28	39	42
Metabolism of aromatic compounds	6	14	10
Miscellaneous	12	22	19
Motility and chemotaxis	0	0	0
Nitrogen metabolism	16	26	22
Nucleosides and nucleotides	49	63	64
Phages, prophages, transposable elements, and plasmids	1	1	1
Phosphorus metabolism	13	17	16
Photosynthesis	0	0	0
Potassium metabolism	11	17	15
Protein metabolism	99	127	166
Regulation and cell signaling	6	16	33
Respiration	24	47	42
RNA metabolism	29	120	121
Secondary metabolism	4	4	4
Stress response	16	48	46
Sulfur metabolism	13	35	27
Virulence, disease, and defense	24	49	44

Strains: 1, *W. gilva* chi5^{T}; 2, *W. fucanilytica* CZ1127^{T}; 3, *W. marina* DSM 100572^{T}.

Table S5. Number of ORFs in the novel strains chi1^T and chi5^T compared with those in related species belonging to the genera *Marinobacter* and *Wenyingzhuangia*, respectively.

(A) Number of ORFs in strain chi1^T and closely related species.

Characteristics	Species		
	1	2	3
Auxiliary activity	7	7	10
Carbohydrate-binding module	0	1	6
Carbohydrate esterase	7	6	1
Glycoside hydrolase	8	13	12
Glycosyl transferase	30	34	42
Polysaccharide lyase	0	1	0
Total ORF number	52	62	71

Strains: 1, *M. suaedae* chi1^T; 2, *M. nitratireducens* AK21^T; 3, *M. salinexigens* ZYF650^T.

(B) Number of ORFs in strain chi5^T and closely associated species.

Characteristics	Species				
	1	2	3	4	5
Carbohydrate-binding module	7	2	13	2	4
Carbohydrate esterase	22	13	14	9	19
Glycoside hydrolase	122	107	145	131	124
Glycosyl transferase	52	47	51	53	53
Polysaccharide lyase	15	5	17	8	10
Total ORF number	218	174	240	203	210

Strains: 1, *W. gilva* chi5^T; 2, *W. aestuarii* DSM 105044^T; 3, *W. fucanilytica* CZ1127^T; 4, *W. heitensis* DSM 101599^T; 5, *W. marina* DSM 100572$^{T.}$

Table S6. Results of pangenomic analysis of the novel strains chi1^T and chi5^T and reference strains belonging to the genera *Marinobacter* and *Wenyingzhuangia*, respectively, using Build Pangenome with OrthoMCL version 2.0.

(A) Pangenomic profiles of strain chi1^T and closely related species.

Genome	Genes	Homologous genes	Singleton genes	Homologous families
M. suaedae chi1^T	3,230	3,033	197	2,969
M. guineae M3B^T	4,130	3,876	254	3,780
M. adhaerens HP15^T	4,410	3,895	515	3,773
M. flavimaris KCTC 12185^T	4,077	3,843	234	3,732
M. sediminum R65^T	3,356	3,209	147	3,166
M. gudaonensis CGMCC 1.6294^T	3,434	3,312	122	3,259
M. algicola DG893^T	4,127	3,817	310	3,655
M. salinus Hb8^T	3,732	3,540	192	3,476
M. lipolyticus SM19^T	3,620	3,458	162	3,370
M. pelagoius HS225^T	3,473	3,326	147	3,257
M. nitratireducens AK21^T	3,491	3,402	89	3,331
M. santoriniensis NKSG1^T	3,690	3,509	181	3,416
M. profrundi PWS21^T	3,605	3,446	159	3,340
M. nauticus ATCC 49840^T	3,614	3,481	133	3,419
M. koreensis DD-M3^T	3,475	3,347	128	3,305
M. salinexigens ZYF650^T	3,855	3,592	263	3,474
M. xestospongiae UST090418-1611^T	4,253	3,573	680	3,442
M. mobilis CGMCC 1.7059^T	3,603	3,420	183	3,184
M. daepoensis DSM 16072^T	3,484	3,257	227	3,206
M. aromaticivorans D15-8P^T	3,734	3,367	367	3,279
M. salarius R9SW1^T	3,168	2,786	382	2,691

B) Pangenomic profiles of strain chi5^T and closely related species.

Genome	Genes	Homologous genes	Singleton genes	Homologous families
W. gilva chi5^T	2,855	2,487	368	2,411
W. aestuarii DSM 105044^T	2,992	2,708	284	2,601
W. fucanilytica CZ1127^T	2,803	2,503	300	2,353
W. heitensis DSM 101599^T	3,071	2,683	388	2,558
W. marina DSM 100572^T	2,758	2,491	267	2,417

Table S7. Cellular fatty acid profiles of strains chi1T and chi5T and closely related reference strains.

(A) Cellular fatty acid profiles of strain chi1T and closely related species.

Fatty acid	Strains		
	1	2	3
$C_{12:0}$	12.1	9.5	12.2
$C_{14:0}$	0.8	2.4	2.9
$C_{16:0}$	23.7	24.7	21.7
$C_{17:0}$	2.1	1.0	2.6
$C_{12:0}$ 3OH	7.5	10.6	14.0
Iso-$C_{17:0}$	1.0	TR	TR
$C_{16:1}$ $\omega5c$	0.8	TR	TR
$C_{17:1}$ $\omega8c$	3.5	1.1	1.7
$C_{18:1}$ $\omega9c$	1.1	19.9	22.0
**Summed feature 3***	31.4	5.3	2.25
**Summed feature 8***	6.7	N/D	N/D
**Summed feature 9***	2.6	−	1.2

Strains: 1, *M. suaedae* chi1T; 2, *M. nitratireducens* AK21T; 3, *M. salinexigens* ZYF650T.

*Summed features are fatty acids that cannot be resolved reliably from other fatty acids using the chosen chromatographic conditions. The MIDI system groups these fatty acids into one feature with a single percentage of the total. Summed feature 3 consists of $C_{16:1}$ $\omega6c$ and/or $C_{16:1}$ $\omega7c$. TR, the amou nt is less than 1% of the total; −, not detected; N/D, no data.

(B) Cellular fatty acid profiles of strain chi5T and closely related species.

Fatty acid	Strains		
	1	3	5
$C_{10:0}$	1.0	−	−
$C_{12:0}$	9.9	−	−
$C_{14:0}$	4.6	−	−
$C_{15:0}$	1.4	−	−
$C_{16:0}$	3.5	3.5	2.2
$C_{18:0}$	1.2	−	−
$C_{16:0}$ 3OH	1.5	3.7	1.5
Iso-$C_{15:0}$ 3OH	11.1	12.2	15.0
Iso-$C_{17:0}$ 3OH	14.7	17.4	15.2

Iso-$C_{13:0}$	6.5	3.8	5.7
Iso-$C_{15:0}$	24.1	17.6	18.0
Iso-$C_{15:1}$ G	6.2	6.3	12.0
**Summed feature 3***	6.2	13.1	10.3

Strains: 1, *W. gilva* chi5^T; 2, *W. fucanilytica* CZ1127^T; 3, *W. marina* DSM 100572^T.

*Summed features are fatty acids that cannot be resolved reliably from other fatty acids using the chosen chromatographic conditions. The MIDI system groups these fatty acids into one feature with a single percentage of the total. Summed feature 3 consists of $C_{16:1}$ *ω6c* and/or $C_{16:1}$ *ω7c*. −, not detecte d.

Table S8. Polar lipids profiles of strains chi1^T and chi5^T and closely related reference strains.

Polar lipids	Strains		
	1	2	3
AGL	1	0	0
AL	0	0	2
APL	0	1	0
DPG	1	1	1
PE	1	1	1
PG	1	1	1
PL	1	2	2
UL	0	1	3

Strains: 1, *M. suaedae* chi1^T; 2, *M. nitratireducens* AK21^T; 3, *M. salinexigens* ZYF650^T.

Polar lipids	Strains		
	4	5	6
AGL	1	0	0
AL	1	3	0
GL	4	0	0
PE	1	1	1
PL	0	1	0
UL	1	10	4

Strains: 1, *W. gilva* chi5^T; 2, *W. fucanilytica* CZ1127^T; 3, *W. marina* DSM 100572^T.

Abbreviations: APL; unidentified aminophospholipid.
